# The macromolecular crystallography beamline I911-3 at the MAX IV laboratory

**DOI:** 10.1107/S0909049513011734

**Published:** 2013-05-18

**Authors:** Thomas Ursby, Johan Unge, Roberto Appio, Derek T. Logan, Folmer Fredslund, Christer Svensson, Krister Larsson, Ana Labrador, Marjolein M. G. M. Thunnissen

**Affiliations:** a MAX IV Laboratory, Lund University, POB 118, Lund SE-221 00, Sweden; b Biochemistry and Structural Biology, Lund University, POB 124, Lund SE-221 00, Sweden

**Keywords:** macromolecular crystallography beamline, anomalous dispersion phasing, beamline automation, crystal dehydration

## Abstract

The macromolecular crystallography beamline I911-3, part of the Cassiopeia/I911 suite of beamlines, is based on a superconducting wiggler at the MAX II ring of the MAX IV Laboratory in Lund, Sweden. The beamline is energy-tunable within a range between 6 and 18 keV. I911-3 opened for users in 2005. In 2010–2011 the experimental station was completely rebuilt and refurbished such that it has become a state-of-the-art experimental station with better possibilities for rapid throughput, crystal screening and work with smaller samples. This paper describes the complete I911-3 beamline and how it is embedded in the Cassiopeia suite of beamlines.

## Abstract 2

The updated macromolecular crystallography beamline I911-3 at the MAX II storage ring is described.

## 1. Introduction   

The MAX IV Laboratory is a Swedish national facility dedicated to research requiring synchrotron light. Its user community is highly international, with a mixed background in physics, chemistry and life sciences.

At present the MAX IV Laboratory houses three storage rings (MAX I, MAX II and MAX III), as well as a free-electron laser (FEL) test facility, and the laboratory is also engaged in the building and development of the new facility MAX IV. The oldest storage ring is MAX I (1985), which operates at 550 MeV (Eriksson, 1982 ▶). MAX II, which became operational in 1997, is a 1.5 GeV ring (Andersson *et al.*, 1994 ▶). The most modern of the three rings is MAX III, which operates at 700 MeV and became operational in 2008 (Sjöström *et al.*, 2009 ▶).

The first beamline on the MAX II storage ring, I711 (Cerenius *et al.*, 2000 ▶), was originally a multipurpose beamline used for both macromolecular and small molecule crystallography as well as powder diffraction experiments. Since it was only manually tunable, a time-consuming process and its relative bandwidth was of the order of 10^−3^, it was not suitable for precise multiple-wavelength experiments. A new suite of beamlines (I911, or Cassiopeia) dedicated to structural biology was developed, based on a liquid-He-cooled superconducting wiggler (Wallén *et al.*, 2001 ▶). Cassiopeia consists of four side-stations (I911-1, 2, 4 and 5) that have fixed wavelength, while the central station I911-3 was built to extend the capabilities of MAX-lab to include facilities for phasing using tunable wavelengths. The four fixed-wavelength side-stations all use optics of similar design (Mammen *et al.*, 2004 ▶, 2005 ▶) and are used for macromolecular crystallography (I911-2 and I911-5), small-angle X-ray scattering (I911-4; Labrador *et al.*, 2013 ▶) and for various other scattering experiments such as grazing-incidence and reflectivity measurements (I911-1; Feidenhans’l, unpublished results).

I911-3 is an energy-tunable automated beamline built for macromolecular crystallography of small crystals (Ursby *et al.*, 2004 ▶). It is tunable in the range 0.7–2.0 Å, with a flux of around 4 × 10^11^ photons s^−1^ at 1 Å. The beamline became available for users in 2005. The experimental hutch was equipped at that time with a Crystal Logic goniostat (Crystal Logic, Los Angeles, USA) combined with a MarMosaic 225 mm area detector (Rayonix, Evanston, USA). In order to automate the station fully and improve the signal-to-noise ratio for small samples, it was decided to redesign and refurbish the experimental hutch. We describe here the upgraded beamline I911-3 and the new experimental environment, summarized in Table 1 ▶.

## 2. Beamline overview   

### 2.1. The MAX II storage ring   

MAX II is a third-generation 1.5 GeV storage ring with 90 m circumference and 9 nm rad horizontal emittance, usually operated with 10% coupling. The ring is injected from a 500 MeV re-circulating linac injector (Andersson *et al.*, 2000 ▶) that also serves MAX I, MAX III and the FEL test facility (Werin *et al.*, 2009 ▶) situated in the centre of MAX II. MAX II is usually injected every 12 h, giving an average current of 200 mA.

### 2.2. Insertion device   

The I911-3 beamline uses a liquid-He-cooled superconducting wiggler as X-ray source to shift the spectrum from MAX II to higher energies. The wiggler has 47 poles with 61 mm period and was designed and built at MAX-lab (Wallén *et al.*, 2001 ▶). It is designed for magnetic fields up to 3.5 T but is usually operated at 2.8 T, giving a critical energy of 4.2 keV and a peak brilliance of 2 × 10^15^ photons s^−1^ (0.1% bandwidth)^−1^ mrad^−2^ mm^−2^. The calculated flux through the primary slits is 1.6 × 10^13^ photons s^−1^ (0.1% bandwidth)^−1^.

### 2.3. The Cassiopeia suite of beamlines and side-station optics   

**Figure 1:**
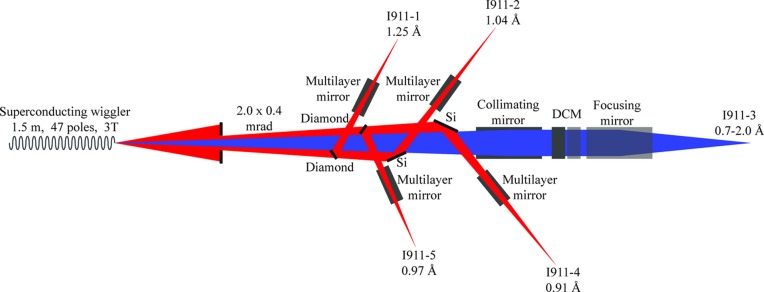
Schematic drawing of the Cassiopeia/I911 optics. The diamond and silicon monochromators of the I911-1, 2, 4 and 5 stations select monochromatic beams (shown in red) for the side-stations. The beam for I911-3 (shown in blue) passes undisturbed through the side-station optics to reach the I911-3 optical elements.

The concept of Cassiopeia was to build a suite of beamlines around the same wiggler source that optimally exploited the wide fan of radiation from the wiggler (Mammen *et al.*, 2004 ▶). Cassiopeia was therefore designed to include four fixed-wavelength side-stations, I911-1, 2, 4 and 5, and one central tunable wavelength station, I911-3 (see Fig. 1 ▶). A fixed aperture in the front-end permits 2 mrad of the wiggler beam fan to pass through. Of this beam fan, the central 0.5 mrad is left unperturbed by the side-station optics and is used for I911-3, while the rest is used for the side-stations.

The optics for each side-station consists of a monochromator crystal followed by a curved multilayer mirror. For the two upstream side-stations (nearest the source; I911-1 and 5) the monochromator crystals are flat 111 diamonds working in Laue geometry. For the two downstream side-stations (I911-2 and 4), bent Si(111) crystals in Bragg geometry are used (Mammen *et al.*, 2004 ▶). All the optical elements for the four side-stations are on a single table (JJ X-Ray, Kgs Lyngby, Denmark).

### 2.4. I911-3 optics   

**Figure 2:**
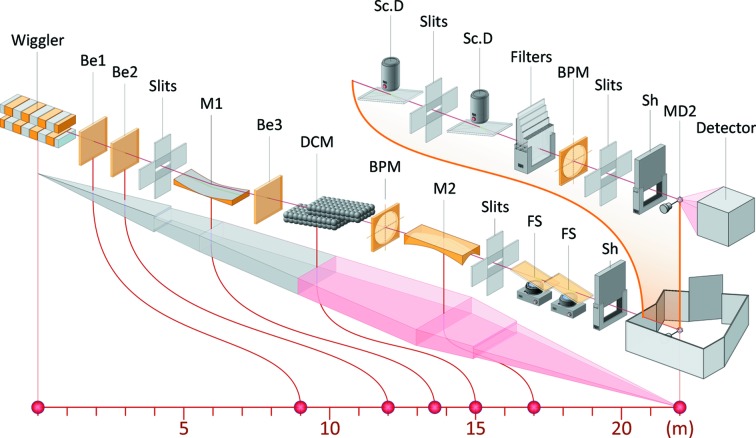
The main components of the I911-3 beamline. The components shown are the wiggler, beryllium windows (Be), slits, mirrors (M1 and M2), double-crystal monochromator (DCM), beam position monitors (BPM), fluorescence screens (FS), shutters (Sh), scintillation detectors (Sc.D), filters, goniostat (MD2) and detector. The upper inset shows components in the experiment hutch, which is shown with schematic walls in the lower figure. The sample changer is not included in the figure. The front-end is situated between the wiggler and the first beryllium window (Be1), and the optics for I911-1, 2, 4 and 5 between Be1 and Be2. Illustration by Johnny Kvistholm.

The optics for I911-3 consists of a collimating mirror (M1), a double-crystal monochromator (DCM) and a focusing mirror (M2) (all by Oxford Danfysik, Oxford, UK, now FMB Oxford, with mirrors and mirror benders delivered by SESO, Aix-en-Provence, France) (see Fig. 2 ▶, which shows the main beamline components). The collimating mirror is a 1.1 m-long Si crystal with a Cr adhesive coating and a Rh surface coating. The mirror is cooled by two water-cooled Ni-coated Cu fins dipping into an InGaSn eutectic bath in two slots in the mirror substrate. The mirror is upwardly reflecting and bent to a radius of curvature of 7.6 km in the meridional direction. The bender is of the U-bender type (Fermé & Dahan, 1998 ▶). The DCM has a first flat crystal (80 mm × 90 mm × 4 mm, length × width × thickness) mounted on a water-cooled Cu block, followed by a second flat crystal (50 mm × 40 mm × 10 mm, length × width × thickness). The original first crystal was an internally water-cooled crystal from Polovodice (now ABB/Polovodice, Prague, Czech Republic), consisting of two crystals soldered together with an aluminium-based material. Owing to repeated problems with leaks between the water circuit and the vacuum, we replaced the internally cooled first crystal by an indirectly cooled crystal. The second mirror is a cylindrically polished Si mirror with the same coating as the first mirror. The radius of curvature in the sagittal direction is 27.6 mm while the mirror is bent in the meridional direction with a radius of curvature of 3.0 km to give a toroidal shape.

Upstream of M1 there are a set of primary slits and a filter unit. The DCM includes a PIN diode (Hamamatsu Photonics, Hamamatsu, Japan) that measures the incoming intensity. Directly after the DCM there is a vertical beam position monitor (FMB Oxford, Oxford, UK) consisting of a thin Ni or Ti foil and four PIN diodes. After M2, two fluorescence screens can be inserted for inspection of the monochromatic beam.

**Figure 3:**
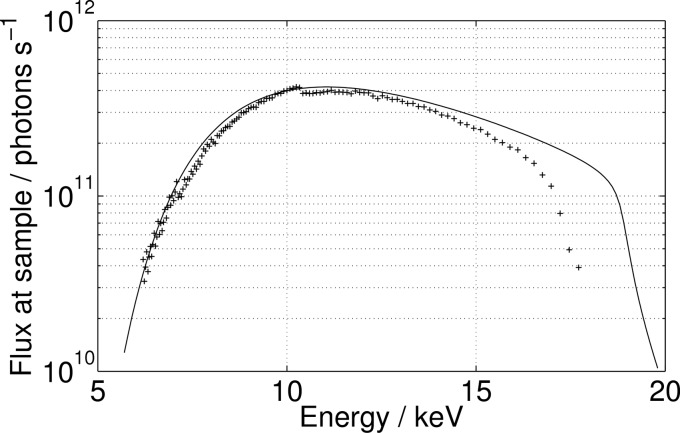
Flux as a function of energy. The markers show the spectrum as measured with a PIN diode at the sample position. The solid line is the theoretical spectrum multiplied by 0.5. The experimentally measured flux is thus a factor of two lower than predicted. The lower flux at high energies is due to the grazing-incidence angles of the mirrors being smaller than the design.

The beamline can be tuned in the range 0.7–2.0 Å though it is usually operated in the range 0.9–1.8 Å. The flux in the focused beam at 1 Å is 4 × 10^11^ photons s^−1^ (see also Table 1 ▶ and Fig. 3 ▶).

### 2.5. Mini-hutches   

Since the floor space available for the five stations is limited, it was decided to keep the experimental hutches at a minimum size. The initial I911-3 enclosure was 2.5 m long, 1.25 m wide and 2.5 m high and made of 3 mm-thick stainless steel. Similar enclosures are used at I911-2 and I911-5, while I911-1 and I911-4 have smaller enclosures. The experiment set-ups can be accessed through sliding windows without the requirement to enter the hutches. For full access in case of, for example, equipment servicing there are removable panels. Since the sliding window gives users fast access to their experiments, it has been a popular feature.

### 2.6. Experimental set-up and hutch at I911-3   

The experimental station of I911-3 was completely rebuilt in 2010 with a new radiation safety hutch and replacement of most of the experimental instrumentation, including goniostat, support tables, sample changer, fluorescence detector and most of the beam-conditioning equipment. The replacements give I911-3 a state-of-the-art set-up for automated data collection and allow very good data to be collected from small crystals.

The beam-conditioning equipment within the experiment hutch upstream of the diffractometer consists of a Cyberstar 1 mm NaI(Tl) scintillation detector with a Cyberstar pulse processing unit (also used as a radiation safety detector) (FMB Oxford, Oxford, UK), a pair of slits (JJ X-Ray, Kgs Lyngby, Denmark), an additional identical scintillation detector, two filter units with in total eight axes (XIA, Hayward, CA, USA), a beam position monitor (Fuchs *et al.*, 2008 ▶), a second pair of slits (JJ X-Ray) and a fast shutter (Uniblitz, Rochester, NY, USA).

The experimental set-up has a MD2 microdiffractometer (Perrakis *et al.*, 1999 ▶) equipped with a mini-kappa goniometer MK3 (both from Maatel, Voreppe, France) having an extended horizontal translation range to allow working with crystallization plates. A five-hole beam-defining aperture (BDA) with cleaning capillary plus beamstop is mounted on the MD2 such that the X-ray beam can be shaped to 30, 50, 75, 120 or 150 µm-diameter size at 26 mm upstream of the sample. This makes it possible to work with small crystals while maintaining a low X-ray background. Even with the largest beam-defining aperture the X-ray background is 10–15 times lower than with the previous set-up.

Samples can be mounted either manually or by the use of the CATS sample changer (Jacquamet *et al.*, 2009 ▶) (Irelec, Saint-Martin-d’Hères, France) that has a capacity of 90 samples in its storage dewar, using EMBL/ESRF pucks with SPINE standard pins and vials (Cipriani *et al.*, 2006 ▶). This ensures that a large number of crystals can be screened easily.

**Figure 4:**
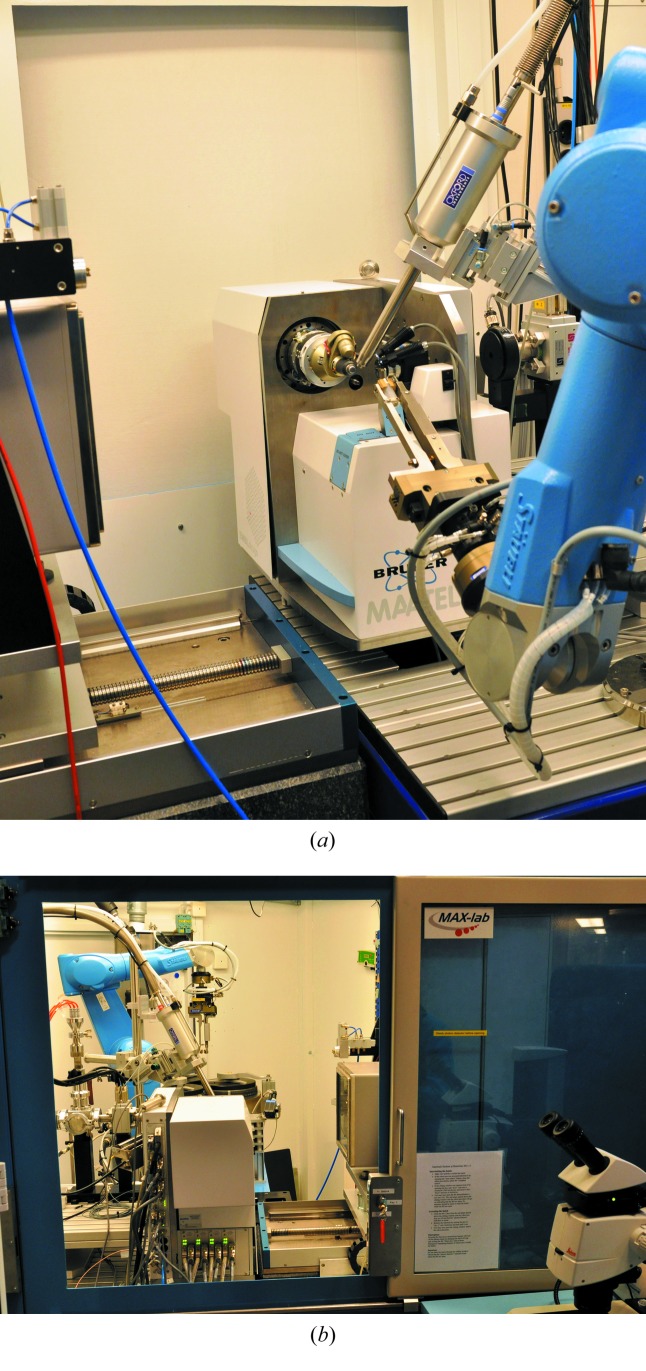
Photographs of the I911-3 experiment set-up. (*a*) The area detector, the MD2 microdiffractometer with the sample cooler, and the sample changer. (*b*) View from the opposite side showing the experiment set-up through the sliding door that is used for manually mounting the samples. When used, the HC1c nozzle replaces the Oxford Instruments sample cooler.

The detector at the station is the original MarMosaic 225 mm area detector (Rayonix, Evanston, IL, USA). The detector can be moved between 55 and 770 mm distance from the sample position. For obtaining the X-ray absorption edge scans required for MAD experiments, a 10 mm^2^ Bruker XFlash fluorescence detector (Bruker-AXS, Berlin, Germany) is available, which can also be used for element analysis using X-ray fluorescence measurements. The samples can be cooled by an Oxford Instruments N_2_ gas sample cooler (Oxford Instruments, Abingdon, UK) to temperatures between <100 K and 300 K. The normal data collection temperature is 100 K. Fig. 4 ▶ shows the experiment set-up.

Since the new experimental set-up required a larger hutch, the enclosure itself was also redesigned and reconstructed. The new hutch is still relatively small, allowing users to mount crystals through a similar sliding window as before with fast access from the sample handling table, which is equipped with a Leica M165C microscope with a Leica IC80 HD video/still camera coupled to a large display. An additional door allows users to enter the hutch for filling the sample-changer storage dewar with their samples.

### 2.7. Experiment control and data analysis   

The beamline hardware is controlled by *spec* (Certified Scientific Software, Cambridge, MA, USA) through a number of different protocols. Most of the optics motors are controlled through a VME rack with OMS VME58 controllers (Oregon Micro Systems, now Pro-Dex OMS Motion Control Products, Irvine, CA, USA) using various Mclennan drivers (Mclennan Servo Supplies Ltd, Ash Vale, UK). The tuning of pitch and roll of the DCM crystals is performed with long-range piezo translations (Newport Corporation, Irvine, CA, USA) and precision piezo translations (piezosystem jena GmbH, Jena, Germany). Experiment table and detector distance and the last slit pair are controlled using Galil DMC-2143 and DMC-4060 controllers (Galil Motion Control, Rocklin, CA, USA). Communication with the MD2 microdiffractometer uses the MD2 Tango device server. The CATS sample-changer communication is performed through TCP/IP. The area detector is controlled by *spec* *via* the MarCCD program that is also used for visualizing the diffraction images that are stored on an internal disk of the detector computer. The data frames are regularly transferred to an 8 TB storage system.

To provide an attractive way to control, plan and execute the experiments, *mxCuBE* (Gabadinho *et al.*, 2010 ▶) is used. The sample changer is currently controlled by a separate GUI provided by the manufacturer Irelec, but can also be controlled through *spec* and work is ongoing to integrate it into the *mxCuBE* GUI. Crystal centering is easily achieved through a three-click procedure in the MD2 GUI.

Data processing is carried out using a separate computer (at present Intel Quad Core CPU Q9550, 2.83 GHz) to ensure that there is no interference with hardware control or data acquisition. The full range of standard macromolecular crystallography software such as *MOSFLM/iMOSFLM* (Leslie & Powell, 2007 ▶), *XDS* (Kabsch, 2010 ▶), *XPREP* (Bruker, Madison, WI, USA), *HKL2MAP* (Pape & Schneider, 2004 ▶), the *CCP4* (Winn *et al.*, 2011 ▶), *SHELX* (Sheldrick, 2008 ▶) and *Phenix* (Adams *et al.*, 2010 ▶) suites are available for data processing and subsequent structure solution. Work is ongoing to implement data processing pipelines such as *EDNA* and *XIA2*. This will also include *BEST* (Bourenkov & Popov, 2006 ▶) to support automatic generation of data collection strategies.

We plan to add ISPyB (Delagenière *et al.*, 2011 ▶) to the beamline for sample tracking, which is becoming increasingly important with increasing number of samples and is a prerequisite for the planned remote operation mode.

## 3. Ancillary facilities   

### 3.1. Humidity control device   

In addition to the set-up described above, the beamline is equipped with an HC1c humidity controller (Sanchez-Weatherby *et al.*, 2009 ▶). The HC1c allows precise control of the humidity environment of protein crystals, which makes it feasible to manipulate the solvent content to improve their diffraction properties. In addition, the HC1c can be used to collect diffraction data from crystals at room temperature without the need for complicated mounting in capillaries that could physically harm them. This allows users to investigate easily the effect of their cryocooling methods and the choice of cryoprotectant on their system. Careful drying of the crystals and working at the appropriate humidity level can in many cases lead to successful crycooling in the absence of a cryprotectant (Russi *et al.*, 2011 ▶). Once the correct conditions are found, the samples are brought to liquid-nitrogen temperature using the sample changer. Experiments using the HC1c need some assistance from the staff; therefore its use needs to be requested by users some weeks in advance.

### 3.2. User laboratory and crystallization facility   

A wet laboratory is available for the users in close proximity to the beamline, shared with the other hard X-ray beamlines I711 and I811. The MAX IV Laboratory also hosts a crystallization screening facility equipped with a Freedom Evo 150 liquid-handling robot (TECAN, Mölndal, Sweden), a Mosquito nanolitre dispenser (TTP Labtech, Melbourn, UK) for preparation of crystallization drops and a Minstrel HT UV plate hotel (Rigaku, Sevenoaks, UK) allowing high-throughput visual and UV imaging of crystallization experiments. The facility also hosts equipment for high-throughput biophysical characterization of proteins, principally as a tool to guide design of crystallization conditions. This equipment consists of a Zetasizer APS plate reader (Malvern, Malvern, UK) for dynamic light-scattering experiments and an Mx3005p qPCR machine (Stratagene, now Agilent, Santa Clara, CA, USA) for thermal stability analysis using differential scanning fluorimetry (Niesen *et al.*, 2007 ▶). The crystallization facility is open for all local and national users, as well as international users upon request. The long-term goal is to integrate the crystallization facility fully with the data collection facilities through the implementation of crystallization plate screening using the CATS robot. The beamline also has access to a cold room for basic manipulation of samples at 277 K.

## 4. Facility access   

The MAX IV Laboratory MX beamlines are open to the general user community through a proposal system with three different access programs: (i) a regular proposal for particular projects valid for a full year, summer to summer; (ii) block allocations for combined groups of projects of several user groups valid for two years; (iii) fast access allocation that can be submitted at any time during the year giving access on a very short time basis for one particular experimental visit.

## 5. Results and highlights   

According to the BioSync database (http://www.biosync.org), between 1998 and 2012, 699 protein structures from the MAX IV Laboratory have been deposited in the Protein Data Bank, with 107 entries coming from I911-3 since the first deposition in 2006. The total number of depositions from the Cassiopeia MX beamlines is 295. In connection, 105 peer-reviewed publications based or partly based on data from I911-3 (or a total of 232 publications from the Cassiopeia MX beamlines) have been published since it opened in 2005.

One of the first publications based on data collected at I911-3 and the first new structure solved at I911-3 by using the MAD method was the structure of the outer membrane porin OmpG (Yildiz *et al.*, 2006 ▶) using the original experimental set-up. A more recent highlight is the *ab initio* structure determination of a DEAH helicase Prp43p (He *et al.*, 2010 ▶), which is involved in the remodeling of RNA. The structure was solved by single anomalous dispersion (SAD) phasing using data collected at the absorption peak of a SeMet variant. The structure showed that Prp43p had homology to DNA helicases. Structural elements, such as a β-hairpin wedging in between domains and a nucleotide binding pocket, could be identified that are important for the mechanism of this protein.

The following highlights are based on data from the refurbished and rebuilt beamline. Shahsavar *et al.* (2012 ▶) determined the structure of a nicotinic acetylcholine receptor from *Lymnaea stagnalis* in complex with a potent antagonist. Suprisingly, this antagonist behaved more like a prototypical agonist, albeit that complementary changes also occurred. As the antagonist is a model compound used in many physiological experiments, this has implications for current models of the mechanisms of agonists and antagonist.

Kellosalo *et al.* (2012 ▶) determined the structure of the native resting form of a sodium pumping pyrophosphatase. This membrane protein is found in plants, protozoans, bacteria and archea and is important for generating an electrochemical potential across the membrane by linking pyrophosphate hydrolysis or synthesis to sodium or proton pumping. It is important for plant development and survival under stress conditions (*e.g.* drought or cold weather). The structure of this protein reveals a new mechanism for the pumping that differs from the mechanisms found in different classes of membrane pumps. In addition to the native data, anomalous data for a Gd derivative were also collected at I911-3. The Gd derivative is an inhibitor for the protein activity and could be used to identify additional metal binding positions.

## 6. Discussion and conclusion   

The MAX IV Laboratory has supported protein crystallography since 1997, when the multi-technique beamline I711 at the MAX II ring opened for users. In recognition of the importance of the MAD and SAD techniques for phasing new crystal structures of proteins, the new suite of beamlines, I911 or Cassiopeia, was developed and constructed. The central beamline is I911-3, suitable for experiments that require a tunable wavelength. In order to supply users with a more automated instrumentation on which smaller crystals can be exposed and large numbers of samples can be screened, the experimental hutch was recently rebuilt and refurbished.

The upgrade of I911-3 has resulted in an improved beamline allowing in particular better data to be collected from small and weakly diffracting crystals, and improved possibilities to screen large number of crystals. One of the advantages with the sample changer is the ability to perform *in situ* diffraction on crystallization plates. This is a valuable tool both in the analysis of protein crystal quality as well as collecting data from multiple crystals at room temperature. Work on integrating this functionality in a GUI is currently ongoing to facilitate its usage. The installation of the new diffractometer and sample-changing robot also prepares I911-3 for user access through remote experimentation.

The MAX IV Laboratory continues its strong support for protein crystallography, as can be seen in BioMAX, one of the seven Phase 1 beamlines at the new MAX IV 3 GeV synchrotron storage ring currently under construction (Thunnissen *et al.*, 2013 ▶). BioMAX will be a dedicated MX beamline and user operations are planned for late 2016. Many of the technical developments implemented at Cassiopeia are directly relevant for the design and construction of BioMAX. Thus the ongoing refurbishments and improvements of Cassiopeia provide valuable experience for BioMAX at the same time as maintaining and developing optimal data collection environments at the present storage ring.

## Figures and Tables

**Table 1:** Main parameters of the I911-3 beamline

Beamline name	I911-3
Source type	Superconducting wiggler ∼3 T, in the MAX II 1.5 GeV storage ring
Mirrors	Rh-coated Si mirrors: M1 collimating mirror, M2 toroidal focusing mirror
Monochromator	Water-cooled double-crystal monochromator, Si(111)
Energy range	6–18 keV
Wavelength range	0.7–2.0 Å
Beam size at sample	0.25 mm × 0.15 mm FWHM (h × v)
Beam divergence at sample	1.7 mrad × 0.7 mrad (h × v)
Flux full beam	4 × 10^11^ photons s^−1^ at 1 Å, 200 mA
Flux, 150 µm BDA	1 × 10^11^ photons s^−1^
Flux, 75 µm BDA	6 × 10^10^ photons s^−1^
Flux, 30 µm BDA	3 × 10^10^ photons s^−1^
Goniostat	MD2 microdiffractometer with MK3 mini-kappa
Sample changer	CATS, 90 samples with EMBL/ESRF standard pucks
Area detector	Marmosaic 225, 225 mm × 225 mm
High-resolution limit at 1 Å	0.95 Å
Fluorescence detector	Bruker/Röntec XFlash 10 mm^2^
